# Efficacy, Safety, and Tolerability of Nasal Wash in Patients with Upper Respiratory Tract Diseases

**DOI:** 10.1055/s-0045-1802576

**Published:** 2025-07-29

**Authors:** Ignazio La Mantia, Giovanna Stilo, Lepanto Lentini, Giorgio Ciprandi

**Affiliations:** 1 Otorhinolaryngology Unit, Azienda Policlinico Catania, Catania, Italy; 2 Department of Otorhinolaryngology, Polo Ospedaliero Umberto I, Siracusa, Italy; 3 Allergy Clinic, Casa di Cura Villa Montallegro, Genova, Italy

**Keywords:** upper respiratory tract diseases, nasal irrigation, saline solution, bicarbonates, PEG

## Abstract

**Introduction:**

Nasal irrigation is a standard therapeutic option to clean the upper airways to remove abundant secretions and harmful substances. The Nasal Wash (SIIT, Milan, Italy) sinus irrigation system contains saline, bicarbonates, and polyethylene glycol.

**Objective:**

The present randomized controlled trial evaluated and compared the Nasal Wash hypertonic solution (NW-HS) with physiological saline (PS) in patients with nasal symptoms common to different upper airway diseases (infectious rhinitis, allergic rhinitis, and acute and chronic rhinosinusitis).

**Methods:**

The symptomatic patients were divided into two groups: those receiving NW-HS 1 to 2 times a day for 7 days and those receiving PS 1 to 2 times a day for 7 days. The primary efficacy measures included total nasal symptom score (TNSS) and the score on the verbal numeric rating scale (VNRS), assessed at baseline and during the treatment period.

**Results:**

In total, 70 patients participated in the trial: 35 were allocated to the NW-HS group and 35 to the PS group. In the NW-HS group, we observed a significant reduction in the TNSS over time, at rates higher than those of the PS group ( *p* < 0.001), as well as a significant reduction in the VNRS score at all observation times, and also at rates higher than those of the PS group ( *p* < 0.001). The safety was good for all patients.

**Conclusion:**

The present trial documented the efficacy and safety of NW-HS in the treatment of nasal symptoms common to upper respiratory tract disorders. Most of the beneficial effects appeared as early as three days after the beginning of the treatment. In addition, compared to PS, NW-HS showed impressive results; thus, it may represent a safe and valuable option in the non-pharmacological therapy for rhinitis.

## Introduction

The nose belongs to the respiratory system and carries out several functions, including: 1) conditioning (by warming, moistening, and filtering) of inspired air; 2) providing adequate innate and adaptive immunity; 3) assuring the olfaction sense; 4) guaranteeing the correct communication with the sinuses and tubaric ostia; and 5) participating in phonation. Since it is a gateway for germs, allergens, pollutants, and harmful agents to enter the body, the nose can quickly become inflammated and/or infected. As a result, an upper respiratory disease occurs. Since the nose is in close communication with other structures connected with the airways (such as the nasopharynx, paranasal sinuses, Eustachian tube, and larynx), when a pathology starts in the nose, it is expected to spread to these different structures, giving rise to various medical conditions, such as nasopharyngitis, rhinosinusitis, laryngitis, and otitis media. All of these diseases are classified differently, depending on their cause and duration; however, they share certain symptoms, including rhinorrhea (runny nose) and nasal congestion/obstruction (stuffy nose), which significantly affect nasal function. In this regard, we must underline that, when the nose is inflammated, its respiration and olfaction functions are significantly impaired, causing hyposmia/anosmia and nasal obstruction. 1 2 3 The removal of harmful substances may be performed through an effective and efficient mechanism called *mucociliary clearance* .

Rhinorrhea is caused by an imbalance in the quantity of mucus in the nasal cavity. The goblet cells continuously produce the mucus layer that covers the mucosa and that is constantly removed by the body; if the body's removal system is impaired or mucus is overproduced because of inflammatory and/or infectious diseases, symptoms occur, and nose functions are impaired. Moreover, mucus stagnation may promote microbial overgrowth, which may be associated with rhinosinusitis, otitis, and the formation of biofilm, leading to recurrent infections. 4 Excessive mucus production may also generate postnasal drip, which triggers a cough.

Nasal obstruction may be due to two leading causes: acute infection and allergy. It results in reduced oxygenation, with potentially relevant systemic consequences, such as hypoxemia and oral breathing, the primary mechanism promoting bronchial involvement, mainly concerning exercise-induced asthma. 5

Consequently, a close and “dirty” nose produces a vicious circle that promotes, maintains, and amplifies respiratory infections and inflammations. 6 As a result, clearing and cleaning the nose are the most simple and helpful remedy that may be pursued in the everyday practice and in patients of all ages. In this regard, nasal lavage is the best way to clean the nose and ensure adequate patency. 7 Nasal lavage works by removing mucus, inflammatory cells, mediators, cytokines, pathogens, and harmful substances form the nasal cavity, cleaning and clearing the nose. Nasal lavage may be performed using different including spray, nasal shower, irrigation, insufflation, fumigation, and aerosol, 8 9 10 as well as different substances (such as pharmacological and non-pharmacological agents) and osmolarity (that is, isotonic or hypertonic solutions).

Nasal irrigation (NI) is currently the most effective method of nasal lavage. 11 12 It is characterized by low-pressure and high-volume fluid delivery, and it assures an optimal distribution of liquids into the nose. The mechanisms of action by which NI works are manifold; it primarily leads to direct cleaning of the nasal mucosa, regardless of the composition of the solution used. The mucus lining the nasal cavity may be softened and dislodged.

Moreover, inflammatory mediators, such as prostaglandins and leukotrienes, as well as antigens responsible for allergic reactions, can be removed, favoring the resolution of upper respiratory tract infections and allergic rhinitis. 13 Nasal irrigation may also be a vehicle to administer medications (medicated NI). Different components are normally used in NI, and the most common is saline solution, which may be isotonic, such as a physiological solution, or hypertonic.

The mechanism of action of saline NI is multifactorial, including direct cleansing of mucus, removal of antigens, biofilm, or inflammatory mediators, and improvement of mucociliary function. 13

Bicarbonates (HCO _3_ ) are also favorably used for NI, normally to buffer hypertonic saline. 14 Polyethylene glycol (PEG) is a polymer of ethylene oxide; commercially, it is most important polyether in terms of production volumes and applications. The physical properties of PEG, such as viscosity, vary with the average length of its macromolecules, that is, the average number of repeating units, while the chemical properties remain virtually unchanged. When it contains few repeating units, PEG is liquid, but, as the number increases, it takes on the appearance of a waxy solid with a relatively low melting point. Polyethylene glycol is soluble in water, methanol, benzene, and dichloromethane, and it is insoluble in diethyl ether and hexane. Polyethylene glycol 3350 is widely used to treat children and adults with constipation; it is an osmotic agent that draws water into the intestinal lumen. 15 In addition, PEG lubricates and maintains the nasal mucosa wet. Therefore, PEG 3350 could be advantageous when added to an NI solution, as it can facilitate the fluidification of mucus, humidify the mucosa, and lubricate the nasal cavity.

In the class of NI products available in the clinical practice, the Nasal Wash (SIIT, Milan, Italy) sinus irrigation system contains saline, HCO _3_ , and PEG, and it product presents the main advantages required to ensure an optimal nasal lavage. 16 Nasal Wash has a large volume that enables a large proportion of the nasal cavities to spread. The system uses only the gravitational force to administer large volumes of the product at low pressure. Indeed, it is well known that low-pressure and high-volume devices are the “gold standard” for nasal delivery. 11 17 Moreover, the possibility of choosing between isotonic and hypertonic solutions enables the treatment to be personalized and modulated over time. Bicarbonate in the isotonic solution helps control the migration of electrolytes through the mucosal wall. In contrast, in the case of the hypertonic solution, the presence of salts helps drain the water from the mucosa, provoking a decongestant and fluidizing effect.

Therefore, the present study has been performed to test this medical device in clinical practice and compare it with the standard physiological saline (PS) solution.

## Methods

Nasal Wash is a class-IIa (Dir. 93/42/EC) medical device developed to treat nasal congestion symptoms, promote nasal mucosa cleansing, prevent secretion accumulation and contamination, and moisturize the irritated mucosa. It is available in sachets containing PEG 3350, sodium chloride (NaCl), and sodium bicarbonate (NaHCO _3_ ) to be dissolved in a bottle containing 240 mL of water. The detailed instructions for reconstituting the product are reported in Table 1 .

**Table 1:** Instructions to prepare Nasal Wash

1) Fill the bottle with drinking water at a temperature of 35–40°C up to the line marking 240 mL.
2) Hypertonic solution: dissolve the contents of two sachets in the bottle. Useful in cases of nasal congestion caused by upper respiratory tract disorders (such as colds, allergic rhinitis, and acute and chronic rhinosinusitis); isotonic solution: dissolve the contents of one sachet in the bottle. Useful in preventing the accumulation of secretions or for daily flushing of the nasal cavities. In both cases, shake the bottle until the powder is completely dissolved in water.
3) Bend your head sideways from the side opposite to the treated nostril and slightly forwards.
4) Insert the end of the bottle into one nostril while breathing with the mouth open.
5) Apply light pressure to the base of the bottle. Wait for the solution to flow into the nose from the opposite nostril.
6) During flushing, breathe in with your mouth open.
7) At the end of the irrigation (half bottle), remain for a few moments with your head bent forward.
8) Repeat the same operation with the other nostril with the remaining half in the bottle.
9) Gently blow your nose.
10) After each use, fill the bottle with water and a few drops of liquid soap, shake vigorously to clean effectively.
11) Dry the inside with a towel or allow it to air dry before next use.

Dissolving 1 sachet in 240 mL of water yields an isotonic solution, while dissolving two sachets yields a hypertonic solution. The dose for the isotonic solution is of one reconstituted bottle up to three times a day, while the hypertonic solution should be administered one to two times a day, depending on clinical severity. Nasal Wash is contraindicated in children under 3 years of age.

### Study Design

In the present study, the Nasal Wash hypertonic solution (NW-HS) was applied to one group of patients and compared with the PS solution (administered to the control group). The patients were randomly (1:1) included on a consecutive basis. In the NW-HS group, each patient performed 1 or 2 nasal irrigations per day, based on the severity of the condition, for 7 consecutive days, reconstituting the product by dissolving 2 sachets in 240 mL of water using the bottle provided. In the control group, each patient performed 1 or 2 nasal irrigations with PS per day, based on the severity of the condition, for 7 consecutive days.

The patients were visited 4 times: at baseline (day 0) and after 3 (day 3), 5 (day 5), and 7 (day 7) days. On days 0 and 7), the patients were evaluated at the office, and on days 3 and 5, they were assessed through telemedicine.

### Patients

The inclusion criteria were subjects aged ≥ 3 years, of both sexes, with nasal symptoms common to different upper airway diseases (such as infectious rhinitis, allergic rhinitis, and acute and chronic rhinosinusitis), total nasal symptom score (TNSS) ≥ 5, and scores ≥ 1 on each measurement parameter.

The exclusion criteria were structural nasal defects (excluded by rhinoscopy), presence of nasal polyps, recent nasal surgery (such as functional endoscopic sinus surgery, FESS), concomitant acute lower respiratory tract disease (pneumonia, bronchitis, bronchiolitis etc.), clinically relevant sleep disorders, pregnancy, breastfeeding, cardiac, respiratory, hepatic, and renal insufficiency, and concomitant treatments that might interfere with outcome measurements (corticosteroids, vasoconstrictors and local anesthetics, antibiotics).

### Tested Products

Nasal Wash consists of a sachet (2.73 g) containing PEG 3350 (400 mg) and salts NaCl (1,675 mg) and NaHCO _3_ (655 mg) to be reconstituted in 240 mL of water to obtain an isotonic (1 sachet; 275–310 mOsm/kg) or hypertonic (2 sachets; 550–620 mOsm/kg) solution for nasal irrigation. The PS solution contained 0.9% of NaCl concentration and was a control treatment.

In the present study, we used the product manufactured under the name Nasal Wash (SIIT, Milan, Italy), but the same product is marketed in Italy (as XNASO) by Levante, Sesto Fiorentino, Italy. In addition, Nasal Wash is marketed by Dr. Max BDC (Prague, Czech Republic) as Dr. Max Nasal Wash Salt in the Czech Republic, Italy, Poland, Romania, and Slovakia. Also, Epsilon Health (Thessaloniki, Greece)"markets Nasal Wash as Nozalys Wash in Greece, Bulgaria, Romania, Serbia, Albania, FYROM, Kosovo, Kuwait, Moldova, and Lebanon.

### Efficacy Measures

The efficacy measures assessed in the current study included nasal symptom scores such as the TNSS and the verbal numeric rating scale (VNRS).

The TNSS is a four-point scale that assesses the severity of the nasal symptoms (0 = absent; 1 = mild; 2 = moderate; and 3 = severe): rhinorrhea, obstruction, itching, irritation/burning, and sneezing. The sum of these five scores is the TNSS.

The VNRS requires the patient to rate their symptomatology perception from 0 to 10, with 0 representing no symptoms, and 10, severe symptoms. While the individual items of the VNRS can be presented vertically or horizontally, the vertical presentation seems to be easier to understand and is often preferred by older patients. Although the use of the visual analog scale (VAS) is acceptable for older patients regarding the psychomotor skills required to complete it, it presents a higher failure rate than other less abstract tools.

The clinical parameters measured by the VNRS were rhinorrhea, nasal obstruction, nasal irritation/burning, dryness of the nasal mucosa, difficulty in falling asleep, waking up during the night, non-restorative sleep, daytime drowsiness, exhaustion-weariness, decreased performance, poor concentration, difficulties in carrying out daily activities at home and at work, difficulty in carrying out social activities (with family, friends, playing with children etc.), and difficulty in carrying out outdoor activities (such as gardening, taking a walk etc.).

### Endpoints

The primary endpoint of the study was to evaluate the effectiveness of NI with Nasal Wash in alleviating nasal symptoms such as rhinorrhea, nasal obstruction, nasal irritation/burning, nasal itching, and sneezing, which are common to several upper respiratory tract diseases (such as infectious and allergic rhinitis, rhinosinusitis, nasopharyngitis), through the TNSS and VNRS.

The secondary endpoints were to compare the efficacy and safety of Nasal Wash with those of the PS solution, to evaluate its efficacy in promoting nasal mucosal hydration by assessing the perception of mucosal dryness and hydration sensation, to assess its safety and tolerability, and to document a possible improvement in sleep quality and quality of life through the VNRS.

### Safety

Throughout the current study, the investigator considered the adverse events related to, possibly related to, or unrelated to the products used.

### Statistical Analysis

The baseline characteristics were expressed as mean and standard deviation values or median and interquartile range values for the continuous variables, while the categorical variables were expressed as absolute and relative frequencies. Due to the non-normal data distribution, the Mann-Whitney U test evaluated differences between the groups at baseline regarding the continuous variables. Associations among the categorical variables were examined using Chi-squared tests.

The Wilcoxon signed-rank test or the Friedman U test were applied to assess differences over time within the NW-HS group. Differences between the groups in terms of delta values (T3–T0) were evaluated using the Mann-Whitney U test. Additionally, we analyzed differences over time (from T0–T3 or from T1–T3) between the groups, and we used repeated measures analysis of variance (ANOVA) to assess the interaction between time and each study group. The results were expressed with two-sided *p* -values. The statistical analysis was conducted using the R (R Foundation for Statistical Computing, Vienna, Austria) software, version 4.1.3.

### Ethical Aspects

The present study was conducted in accordance with the good clinical practice (GCP) standard using the guidelines and practices offered by the International Conference on Harmonization (ICH), European Directive 2001/20/CE, and ISO 14155:2020, and in compliance with the ethical principles of the Declaration of Helsinki (2013), as well as local regulations.

The institutional Ethics Committee approved the protocol (under number 1839) on September 7th, 2023.

## Results

**Fig. 1:**
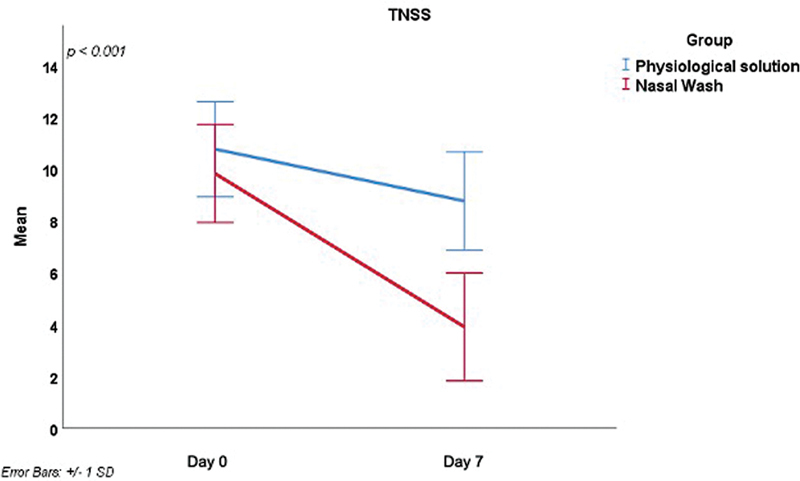
Intergroup comparison of the total nasal symptom score (TNSS) on days 0 and 7.

**Fig. 2:**
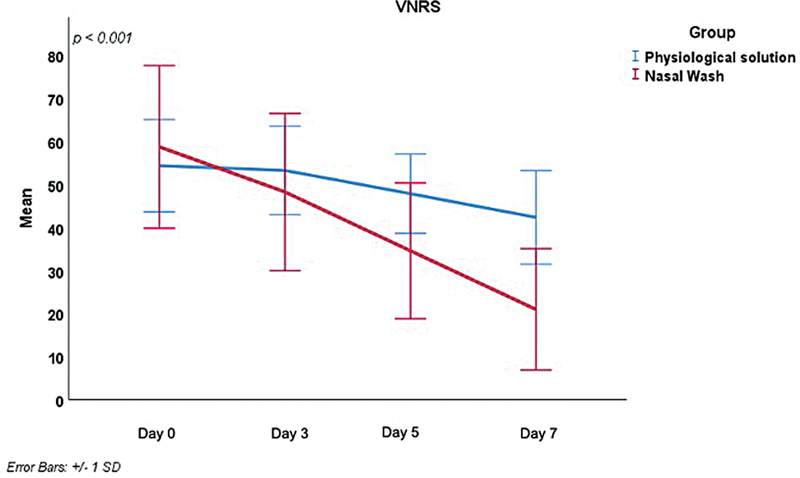
Intergroup comparison of the score on the verbal numeric rating scale (VNRS) on days 0 and 7.

At baseline ( Table 2 ), the 2 groups were well balanced, with a statistical difference observed only for the TNSS, which was higher in the PS group ( *p* = 0.031). Table 3 presents the results of the change in TNSS over time in the NW-HS group. For all nasal symptoms assessed, there was a significant improvement in the partial scores from baseline to posttreatment ( *p* < 0.001). In the group comparison, the mean change in TNSS from baseline to day 7 ( Table 4 ) was of −2.0 ± 0.97 (median: −2.0) in the PS group, and of −5.9 ± 1.56 (median: −6.0) in the NW-HS group, with a significant difference between them ( *p* < 0.001), as reported in Fig. 1 . Table 5 presents the results of the change in VNRS over time in the NW-HS group: there was a significant improvement in the partial scores on all items from baseline to posttreatment ( *p* < 0.001). Significant differences in the VNRS score over time ( Table 6 ) were found between the two groups ( *p* < 0.001). Initially, both presented similar scores ( *p* = 0.55); however, following treatment (day 3), the PS group scored higher on the VNRS than the NW-HS group, a trend that persisted across all time points (days 5 and 7). The intergroup comparison of the VNRS scores at days 0 and 7 showed a significant difference in the NW-HS group ( *p* < 0.001), as reported in Fig. 2 .

**Table 2:** Baseline characteristics of the study sample

		Total	PS group	NW-HS group	*p*
Sex	Female	28 (40.0%)	13 (37.1%)	15 (42.9%)	0.81
	Male	42 (60.0%)	22 (62.9%)	20 (57.1%)	
Mean age (years)		41.6 ± 17.77	40.9 ± 19.23	42.3 ± 16.44	0.75
TNSS: mean ± SD; median (IQR)		10.3 ± 1.91; 10.0 (6.0–15.0)	10.8 ± 1.83; 11.0 (7.0–15.0)	9.8 ± 1.89; 10.0 (6.0–14.0)	0.031*
VNRS score mean ± SD; median (IQR)		56.6 ± 15.42; 54.0 (27.0–95.0)	54.4 ± 10.73; 52.0 (34.0–88.0)	58.8 ± 18.91; 54.0 (27.0–95.0)	0.55

**Abbreviations:** IQR, interquartile range; NW-HS, Nasal Wash hypertonic solution; PS, physiological saline; SD, standard deviation; TNSS, total nasal symptom score; VNRS, verbal numeric rating scale.

Note: *Statistically significant.

**Table 3:** Total nasal symptom score over time in the NW-HS group

	Day 0	Day 7	Delta	*p*
Rhinorrhoea: mean ± SD; median (IQR)	1.9 ± 0.80; 2.0 (1.0–3.0)	1.2 ± 0.75; 1.0 (0.0–3.0)	−0.8 ± 0.77; −1.0 (−2.0–0.0)	< 0.001*
Nasal obstruction: mean ± SD; median (IQR)	2.6 ± 0.55; 3.0 (1.0–3.0)	1.1 ± 0.51; 1.0 (0.0–2.0)	−1.5 ± 0.56; −2.0 (−2.0–0.0)	< 0.001*
Nasal irritation/burning: mean ± SD; median (IQR)	1.9 ± 0.83; 2.0 (1.0–3.0)	0.3 ± 0.48; 0.0 (0.0–1.0)	−1.5 ± 0.89; −1.0 (−3.0–0.0)	< 0.001*
Nasal itching: mean ± SD; median (IQR)	1.6 ± 0.70; 1.0 (1.0–3.0)	0.5 ± 0.70; 0.0 (0.0–2.0)	−1.0 ± 0.57; −1.0 (−2.0–0.0)	< 0.001*
Sneezing: mean ± SD; median (IQR)	1.8 ± 0.68; 2.0 (1.0–3.0)	0.8 ± 0.65; 1.0 (0.0–2.0)	−1.0 ± 0.62; −1.0 (−3.0–0.0)	< 0.001*
TNSS: mean ± SD; median (IQR)	9.8 ± 1.89; 10.0 (6.0–14.0)	3.9 ± 2.08; 3.0 (1.0–10.0)	−5.9 ± 1.56; −6.0 (−9.0–−2.0)	< 0.001*

**Abbreviations:** IQR, interquartile range; NW-HS, Nasal Wash hypertonic solution; SD, standard deviation; TNSS, total nasal symptom score.

Note: *Statistically significant.

**Table 4:** Total nasal symptom score over time in the two study groups

	PS group	NW-HS group	*p*
Day 0: mean ± SD; median (IQR)	10.8 ± 1.83; 11.0 (7.0–15.0)	9.8 ± 1.89; 10.0 (6.0–14.0)	
Day 7: mean ± SD; median (IQR)	8.8 ± 1.90; 8.0 (6.0–13.0)	3.9 ± 2.08; 3.0 (1.0–10.0)	
∆ _3-0_ : mean ± SD; median (IQR)	−2.0 ± 0.97; −2.0 (−4.0–0.0)	−5.9 ± 1.56; −6.0 (−9.0–−2.0)	< 0.001*

**Abbreviations:** IQR, interquartile range; NW-HS, Nasal Wash hypertonic solution; PS, physiological saline; SD, standard deviation; VNRS, verbal numeric rating scale.

Note: *Statistically significant.

**Table 5:** Scores on the VNRS over time in the NW-HS group

	Day 0	Day 3	Day 5	Day 7	*p*
Rhinorrhoea: mean ± SD; median (IQR)	6.5 ± 2.63; 7.0 (2.0–10.0)	5.5 ± 2.66; 5.0 (1.0–10.0)	4.5 ± 2.32; 4.0 (1.0–10.0)	3.2 ± 2.20; 3.0 (0.0–9.0)	< 0.001*
Nasal obstruction: mean ± SD; median (IQR)	8.5 ± 1.69; 9.0 (3.0–10.0)	6.9 ± 1.87; 7.0 (2.0–10.0)	4.9 ± 1.78; 5.0 (2.0–9.0)	3.1 ± 1.56; 3.0 (1.0–7.0)	< 0.001*
Nasal irritation/burning: mean ± SD; median (IQR)	6.1 ± 3.03; 6.0 (0.0–10.0)	4.6 ± 2.70; 5.0 (0.0–10.0)	3.2 ± 2.03; 4.0 (0.0–7.0)	1.3 ± 1.38; 1.0 (0.0–4.0)	< 0.001*
Dryness of nasal mucosa: mean ± SD; median (IQR)	5.6 ± 2.53; 6.0 (1.0–10.0)	4.1 ± 2.35; 4.0 (0.0–10.0)	2.6 ± 1.75; 2.0 (0.0–7.0)	1.0 ± 1.06; 1.0 (0.0–3.0)	< 0.001*
Difficulty falling asleep: mean ± SD; median (IQR)	5.6 ± 2.31; 6.0 (0.0–9.0)	4.6 ± 2.09; 5.0 (0.0–8.0)	3.3 ± 1.88; 3.0 (0.0–8.0)	2.0 ± 1.62; 2.0 (0.0–6.0)	< 0.001*
Waking up during the night: mean ± SD; median (IQR)	4.1 ± 2.41; 4.0 (0.0–9.0)	3.5 ± 1.92; 3.0 (0.0–7.0)	2.3 ± 1.70; 2.0 (0.0–6.0)	1.4 ± 1.68; 1.0 (0.0–5.0)	< 0.001*
Non-restorative sleep: mean ± SD; median (IQR)	3.5 ± 2.24; 3.0 (0.0–8.0)	2.9 ± 2.19; 3.0 (0.0–8.0)	2.2 ± 1.88; 2.0 (0.0–6.0)	1.3 ± 1.59; 1.0 (0.0–5.0)	< 0.001*
Daytime sleepiness: mean ± SD; median (IQR)	2.4 ± 2.28; 2.0 (0.0–8.0)	2.1 ± 2.03; 1.0 (0.0–8.0)	1.6 ± 1.74; 1.0 (0.0–5.0)	1.2 ± 1.47; 1.0 (0.0–5.0)	< 0.001*
Exhaustion: mean ± SD; median (IQR)	2.4 ± 2.64; 1.0 (0.0–9.0)	2.0 ± 2.16; 1.0 (0.0–7.0)	1.4 ± 1.59; 1.0 (0.0–5.0)	0.9 ± 1.27; 0.0 (0.0–4.0)	< 0.001*
Reduced productivity: mean ± SD; median (IQR)	2.9 ± 2.57; 2.0 (0.0–9.0)	2.6 ± 2.36; 2.0 (0.0–8.0)	1.7 ± 1.83; 1.0 (0.0–6.0)	1.2 ± 1.45; 1.0 (0.0–5.0)	< 0.001*
Poor concentration: mean ± SD; median (IQR)	3.0 ± 2.68; 2.0 (0.0–9.0)	2.7 ± 2.33; 2.0 (0.0–8.0)	1.8 ± 1.84; 1.0 (0.0–6.0)	1.3 ± 1.46; 1.0 (0.0–5.0)	< 0.001*
Daily activities: mean ± SD; median (IQR)	2.9 ± 2.42; 2.0 (0.0–10.0)	2.3 ± 1.84; 2.0 (0.0–7.0)	1.7 ± 1.64; 1.0 (0.0–5.0)	1.0 ± 1.15; 1.0 (0.0–4.0)	< 0.001*
Social activities: mean ± SD; median (IQR)	2.6 ± 2.06; 2.0 (0.0–7.0)	2.2 ± 1.88; 2.0 (0.0–7.0)	1.6 ± 1.56; 1.0 (0.0–5.0)	1.0 ± 1.24; 1.0 (0.0–4.0)	< 0.001*
Outdoor activities: mean ± SD; median (IQR)	2.7 ± 2.15; 2.0 (0.0–7.0)	2.4 ± 1.96; 2.0 (0.0–7.0)	1.7 ± 1.73; 1.0 (0.0–6.0)	1.1 ± 1.49; 1.0 (0.0–6.0)	< 0.001*
Total VNRS score: mean ± SD; median (IQR)	58.8 ± 18.91; 54.0 (27.0–95.0)	48.3 ± 18.29; 42.0 (23.0–92.0)	34.6 ± 15.77; 31.0 (12.0–73.0)	21.0 ± 14.11; 17.0 (4.0–65.0)	< 0.001*

**Abbreviations:** IQR, interquartile range; NW-HS, Nasal Wash hypertonic solution; SD, standard deviation; VNRS, verbal numeric rating scale.

Note: *Statistically significant.

**Table 6:** Score on the VNRS over time in the two study groups

	PS group	NW-HS group	*p*
Day 0: mean ± SD; median (IQR)	54.4 ± 10.73; 52.0 (34.0–88.0)	58.8 ± 18.91; 54.0 (27.0–95.0)	Time*group: < 0.001;* group: 0.005; time: < 0.001;* day 0 versus day 3: < 0.001;* day 0 versus day 5: < 0.001;* day 0 versus day 7: < 0.001;* day 3 versus day 5: < 0.001;* day 3 versus day 7: < 0.001;* day 5 versus day 7: < 0.001*
Day 3: mean ± SD; median (IQR)	53.3 ± 10.31; 51.0 (33.0–81.0)	48.3 ± 18.29; 42.0 (23.0–92.0)	
Day 5: mean ± SD; median (IQR)	47.9 ± 9.20; 46.0 (28.0–68.0)	34.6 ± 15.77; 31.0 (12.0–73.0)	
Day 7: mean ± SD; median (IQR)	42.4 ± 10.86; 41.0 (20.0–71.0)	21.0 ± 14.11; 17.0 (4.0–65.0)	
∆ _3-0_ : mean ± SD; median (IQR)	−12.0 ± 6.18; −12.0 (−25.0–0.0)	−37.8 ± 14.58; −38.0 (−78.0–−11.0)	< 0.001*

**Abbreviations:** IQR, interquartile range; NW-HS, Nasal Wash hypertonic solution; PS, physiological saline; SD, standard deviation; VNRS, verbal numeric rating scale.

Note: *Statistically significant.

**Fig. 3:**
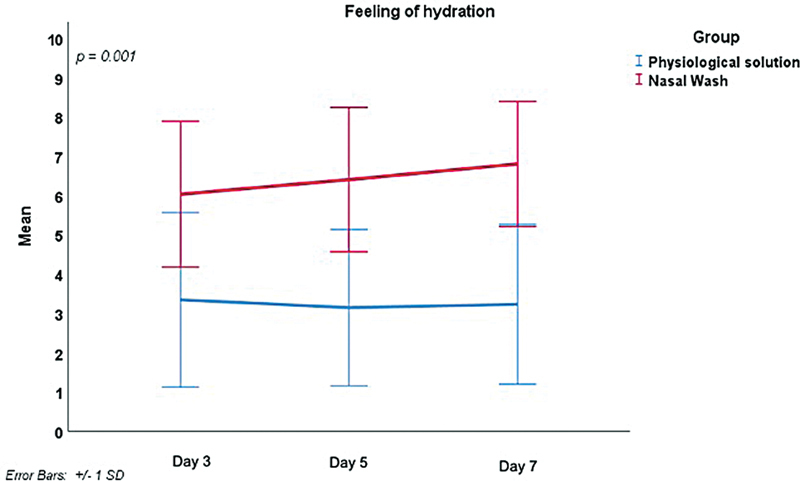
Intergroup comparison of the feeling of hydration on days 0 and 7.

**Fig. 4:**
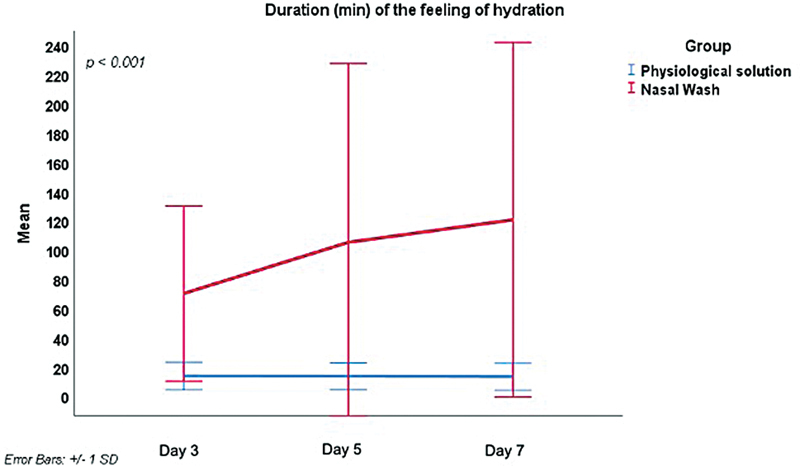
Intergroup comparison of the duration (in minutes) of the feeling of hydration on days 0 and 7.

The feeling of hydration over time significantly differed between the 2 groups ( *p* = 0.001), as reported in Fig. 3 . On day 3, the NW-HS group reported a significantly higher feeling of hydration than the PS group (mean: 6.0 ± 1.85 and 3.3 ± 2.22 respectively), which persisted through days 5 and 7. Likewise, the duration (in minutes) of the feeling of hydration differed significantly over time between the 2 groups ( *p* < 0.001), as reported in Fig. 4 : on day 3, it was significantly longer the NW-HS compared to the PS group (mean: 70.4 ± 60.03 minutes and 13.9 ± 9.43 minutes respectively), a trend observed throughout days 5 and 7.

## Discussion

Upper respiratory tract disorders, including rhinitis, rhinosinusitis, otitis, and nasopharyngitis, are extremely common. Their shared pathophysiological characteristic is the inflammatory reaction that may derive from allergic mechanisms, infectious agents, trauma, surgery, neurological disorders, hormonal dysfunctions etc. Inflammation of the nasal cavity causes the symptoms, and their severity is related to their intensity. In cases of rhinitis, overproduction of mucus is a typical phenomenon that determines the limitation to nasal airflow, and this mucus overflow is a reservoir of mediators, cytokine, microbes, pollen, and pollutants that may induce rhinobronchial syndrome, which is characterized by productive cough.

Thus, removing excessive mucus is the first step in the management of patients with rhinitis despite the pathogenetic mechanism. In this regard, nasal irrigation results in complete lavage of the nasal cavity, assuring the removal of damaging substances. After irrigation, patients usually perceive a pleasant feeling of an open nose and refreshed airways. 6

The present trial compared Nasal Wash, a sinus irrigation system containing salts with HCO _3_ and PEG, with a PS solution. The results confirmed the clear effectiveness of the NW-HS *per se* and compared to a PS solution. Moreover, the presence of PEG and HCO _3_ provided additional benefits.

In particular, NW-HS significantly reduced the global assessment of symptoms (VNRS) as early as the first observation (day 3) compared with PS. In addition, this positive effect persisted and increased progressively over time.

Regarding the symptom assessment by the TNSS, NW-HS significantly reduced symptom severity compared with PS, confirming its efficacy in relieving symptoms more effectively.

In detail, considering the feeling of hydration of the nasal cavity over time, NW-HS significantly increased the sensation of a well-hydrated mucosa at the end of the treatment; on the contrary, the PS did not affect this feeling of hydration. This difference is crucial as it reflects the relevance of PEG as a moisturizing agent. Consistently, the duration of the feeling of hydration significantly increased over time, almost doubling after one week: the sensation of a well-hydrated mucosa went from 70 minutes to more than 2 hours. On the other hand, PS did not modify this sensation; it lasted only 13 minutes, and it was brief. Considering the perception of rhinorrhea over time, NW-HS significantly reduced rhinorrhea severity.

The same results were obtained for the perception of nasal obstruction: NW-HS reduced by more than 60% the perceived intensity of this symptom. Consistently, the mean VNRS score after 1 week of NW-HS treatment was almost reduced to a third (from 8.5 to 3.1). This outcome is clinically relevant, as nasal obstruction is a very annoying symptom that affects quality of life, impairs sleep quality, and reduces oxygenation.

Regarding nasal irritation/burning over time, the mean score for this perception was significantly reduced to about one-quarter. As for the difficulty in going to sleep over time, the NW-HS decreased this complaint as early as the first observation and progressively reduced this drawback by nearly one-third.

Considering another parameter of sleep quality, non-restorative sleep over time, the NW-HS reduced its perception as early as the first observation, and progressively improved this aspect of sleep quality. Poor sleep consistently affects daytime attention and efficiency; the NW- HS reduced daytime sleepiness and improved this aspect at the end of the treatment.

Moreover, impaired sleep determines the sensation of exhaustion and reduced productivity. In this regard, the NW- HS reduced the feeling of exhaustion and improved productivity over time. It also improved concentration, the performance of daily activities, and effectively improved the performance of social and outdoor activities over time. Finally, the safety was excellent, as the use of the NW-HS was well tolerated and did not result in clinically relevant adverse reactions.

However, the present study had some limitations, including the fact that we only evaluated HS reconstitution, without an assessment of biomarkers, the provision of functional data, and with a short follow-up.

## Conclusion

Rhinosinusal diseases, including rhinitis and rhinosinusitis, are common medical conditions affecting nearly every person throughout their lives. Rhinitis is an inflammation of the nasal cavity that frequently involves neighboring tissues, including the sinus, nasopharynx, and internal ear. Rhinitis may present different etiopathogenetic mechanisms; however, the symptoms are substantially the same in all rhinitis phenotypes.

Rhinosinusitis is an inflammatory condition involving the nose and paranasal sinuses that shares the same pathophysiological mechanisms as those of rhinitis. Relieving symptoms remains the first step in managing patients with rhinosinusal diseases.

The pharmacological treatment of rhinosinusal disorders is usually effective, but it can be unsatisfactory in some patients and associated with side effects. Moreover, mucus overflow is unlikely to regress after the use of medications. Thus, nasal irrigation using high-volume and low-pressure devices represents a fruitful option to manage rhinitis patients. Different substances can be used; however, HSs are particularly effective, as they actively remove mucus and decongest inflammated mucosa.

The NW is a sinus irrigation system containing NaCL, HCO _3_ , and PEG. These components exert relevant effects, including mechanical removal, moisturizing, decongestant, and anti-inflammatory properties.

The present trial documented the efficacy and safety of NW-HS in treating nasal symptoms common to upper respiratory tract disorders. Most of the beneficial effects appeared as early as three days after treatment. In addition, NW, compared to the PS solution, showed impressive results.

Thus, NW-HS may represent a safe and valuable option in non-pharmacological rhinitis therapy.
